# Rational design, synthesis, and pharmacological characterisation of dicarbonyl curcuminoid analogues with improved stability against lung cancer via ROS and ER stress mediated cell apoptosis and pyroptosis

**DOI:** 10.1080/14756366.2022.2116015

**Published:** 2022-08-29

**Authors:** Tao Wei, Zhiwei Zheng, Xiaoyan Wei, Yugang Liu, Wentao Li, Bingqing Fang, Di Yun, Zhaojun Dong, Baozhu Yi, Wulan Li, Xiaoping Wu, Dezhi Chen, Liping Chen, Jianzhang Wu

**Affiliations:** a Department of Gynecology and Obstetrics, The Second Affiliated Hospital and Yuying Children’s Hospital of the Wenzhou Medical University, Wenzhou, China; b School of Pharmaceutical Sciences, Wenzhou Medical University, Wenzhou, China; c Oujiang Laboratory (Zhejiang Lab for Regenerative Medicine, Vision and Brain Health), Wenzhou, China; d The Cancer Hospital of the University of Chinese Academy of Sciences (Zhejiang Cancer Hospital), Institute of Basic Medicine and Cancer (IBMC), Chinese Academy of Sciences, Hangzhou, Zhejiang, China; e The First Affiliated Hospital of Wenzhou Medical University, Wenzhou, China; f MOE Key Laboratory of Tumor Molecular Biology, Guangdong, China; g Zhejiang University School of Medicine Sir Run Run Shaw Hospital, Hangzhou, China; h The Eye Hospital of Wenzhou Medical University, Wenzhou, China

**Keywords:** Dicarbonyl curcumin analogues, stability, density functional theory, pyroptosis, anti-lung cancer activity

## Abstract

Curcumin is a natural medicine with a wide range of anti-tumour activities. However, due to β-diketone moiety, curcumin exhibits poor stability and pharmacokinetics which significantly limits its clinical applications. In this article, two types of dicarbonyl curcumin analogues with improved stability were designed through the calculation of molecular stability by density functional theory. Twenty compounds were synthesised, and their anti-tumour activity was screened. A plurality of analogues had significantly stronger activity than curcumin. In particular, compound B2 ((2E,2′E)-3,3′-(1,4-phenylene)bis(1-(2-chlorophenyl)prop-2-en-1-one)) exhibited excellent anti-lung cancer activity *in vivo* and *in vitro*. In addition, B2 could upregulate the level of reactive oxygen species in lung cancer cells, which in turn activated the endoplasmic reticulum stress and led to cell apoptosis and pyroptosis. Taken together, curcumin analogue B2 is expected to be a novel candidate for lung cancer treatment with improved chemical and biological characteristics.

## 1. Introduction

Lung cancer is a malignant tumour that seriously affects human health and life. According to WHO, World Cancer Report 2020, lung cancer accounts for 11.4% of all cancer cases, ranking second. But the death rate still tops the list at 18.0%. The main reason is the lack of typical symptoms, most of which are late-stage cancer when found, missing the best treatment period^1^. Chemotherapy remains one of the main treatments for advanced lung cancer^2^. However, traditional chemotherapeutic drugs such as cisplatin have non-specific targeting, high toxicity and side effects, which limit their further application. Therefore, it is necessary to find new effective chemical drugs. Natural products have the characteristics of wide efficacy and low toxicity, which has become a research hotspot as lead compounds in recent years.

Curcumin, a natural compound separated from the rhizomes of Curcuma longa (isolated 200 years ago), possesses multi-functional pharmacological activity^3^. Congregate research from *in vitro* and pre-clinical studies strongly supports its immense potential as an anti-proliferative, anti-invasive, and anti-angiogenic agent, and it also could enhance chemotherapy or radiotherapy effect^4^^,^^5^. The safety, tolerability, and non-toxicity of this compound at high doses also have been demonstrated by several human clinical trials^6^. However, due to the drug defects caused by its poor stability^6^, more than 274 clinical trials of curcumin have been unsuccessful since 1996 (https://clinicaltrials.gov/). The structural modification thus is one of the major approaches to improve its stability and bioavailability. It is found that the structure of β-diketone group is the mainspring of its *in vivo* less absorption, rapid metabolism and low bioavailability^7–9^. The β-diketone group could accept proton to be enolized (keto-enol tautomerism) by the change of external environment, which associated with the degradation and reduction of curcumin, whereas its binding is mainly via hydroxyl groups^10^^,^^11^. For such reasons, many scholars have focussed on the study of modifying β-diketone group, and lots of monocarbonyl curcumin analogues with improved stability and enhanced activity were designed for the deletion of carbonyl group from β-diketones^12–18^. To date, nevertheless, there are few reports about the dicarbonyl curcumin analogues with improved stability and activity in case of retaining carbonyl groups. Therefore, we inserted a benzene ring into the dicarbonyl structure of curcumin to improve its stability. Although Evelyn Winter et al. designed similar structures of bischalcone ABCG2 inhibitors from chromones, they did not study the stability of the structures^19^. This article showed that the stability of the newly designed compound was significantly higher than that of curcumin. Among all compounds, B2 was shown stronger *in vitro* and *in vivo* anti-tumour activity than that of curcumin. The mechanism of B2 as an anti-lung cancer candidate was also studied that it induced cell apoptosis to pyroptosis by regulating reactive oxygen species (ROS) and activating endoplasmic reticulum (ER) stress.

## 2. Results

### 2.1. Design and stability of dicarbonyl curcumin analogues

**Figure 1.**
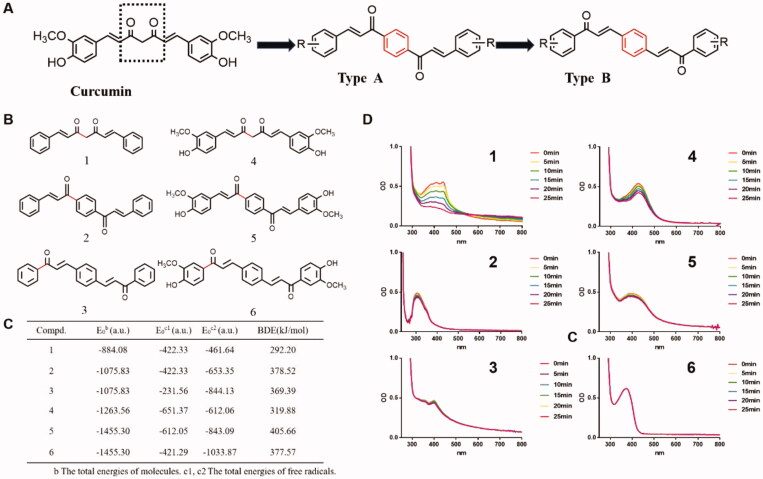
The improved stability of dicarbonyl curcumin analogues A and B. (A) Design of dicarbonyl curcumin analogues A and B. (B) The structure of curcumin skeleton 1, dicarbonyl curcumin skeleton 2, 3, curcumin 4, dicarbonyl curcumin 5, 6. (C) Calculation of BDE of six compounds and total energies (*E*_0_^b^) of molecules and two free radicals (*E*_0_^c1^, *E*_0_^c^^2^) at the B3LYP/6-31G(d) level. (D) The stability of six compounds in PBS solution was determined by UV spectrophotometry (40 mM, per 5 min for 25 min, 250–800 nm).

In this article, two classes of dicarbonyl curcumin analogues A and B were designed (Figure 1(A)). The physical calculation of the bond dissociation was then performed to predict their molecular stability. In this experiment, compounds **1**–**6** were chosen for the prediction of molecular stability, and their structure were showed in Figure 1(B). Compounds **4** and **1** were curcumin and its skeleton respectively, compounds **2** and **3** were the backbone of two classes of dicarbonyl curcumin analogues, and compounds **5** and **6** both were dicarbonyl curcumin analogues with same side chain groups. For the theory that the lower of the bond dissociation energy (BDE), the more unstable of the compound and its chemical bond, the BDE of the compounds and total energies (*E*_0_^b^) of molecules and two free radicals (*E*_0_^c1^, *E*_0_^c^^2^) at the B3LYP/6-31G(d) level thus were calculated. It was showed that the total energy (*E*_0_^b^) of compounds **5** and **6**, which both with a benzene ring structure were −1455.30 au, while the *E*_0_^b^ of curcumin (compound **4**) was −1263.56 au. Besides, the BDE of the most easily broken chemical bond (red) of curcumin was 319.88 kJ/mol, which increased to 405.66 kJ/mol and 377.57 kJ/mol of compounds **5** and **6**, respectively, indicating that the introduction of benzene ring could significantly enhance the stability (Figure 1(C)). The results in Figure 1(C) also indicated that the introduction of substituent in the molecule structure decreased the *E*_0_^b^ and increased the BDE level, which suggested that substituent introduction was conducive to enhancing the stability of compounds. To further test the stability of these compounds, ultraviolet spectra assay was carried out. It was found that the absorption intensity of curcumin and its skeleton in PBS solution decreased significantly with time, and the decreased level of curcumin skeleton was significantly quicker than that of curcumin. While, the newly designed Compounds **2**, **3**, **5**, and **6** had relatively stable absorption wavelengths and did not change significantly with time (Figure 1(D)). Because the stability of the compounds in the PBS solution is consistent with the calculation of the BDE, this indicates that the dicarbonyl curcumin analogues have a more stable structure compared to curcumin.

### 2.2. Chemical synthesis

**Figure 2.**
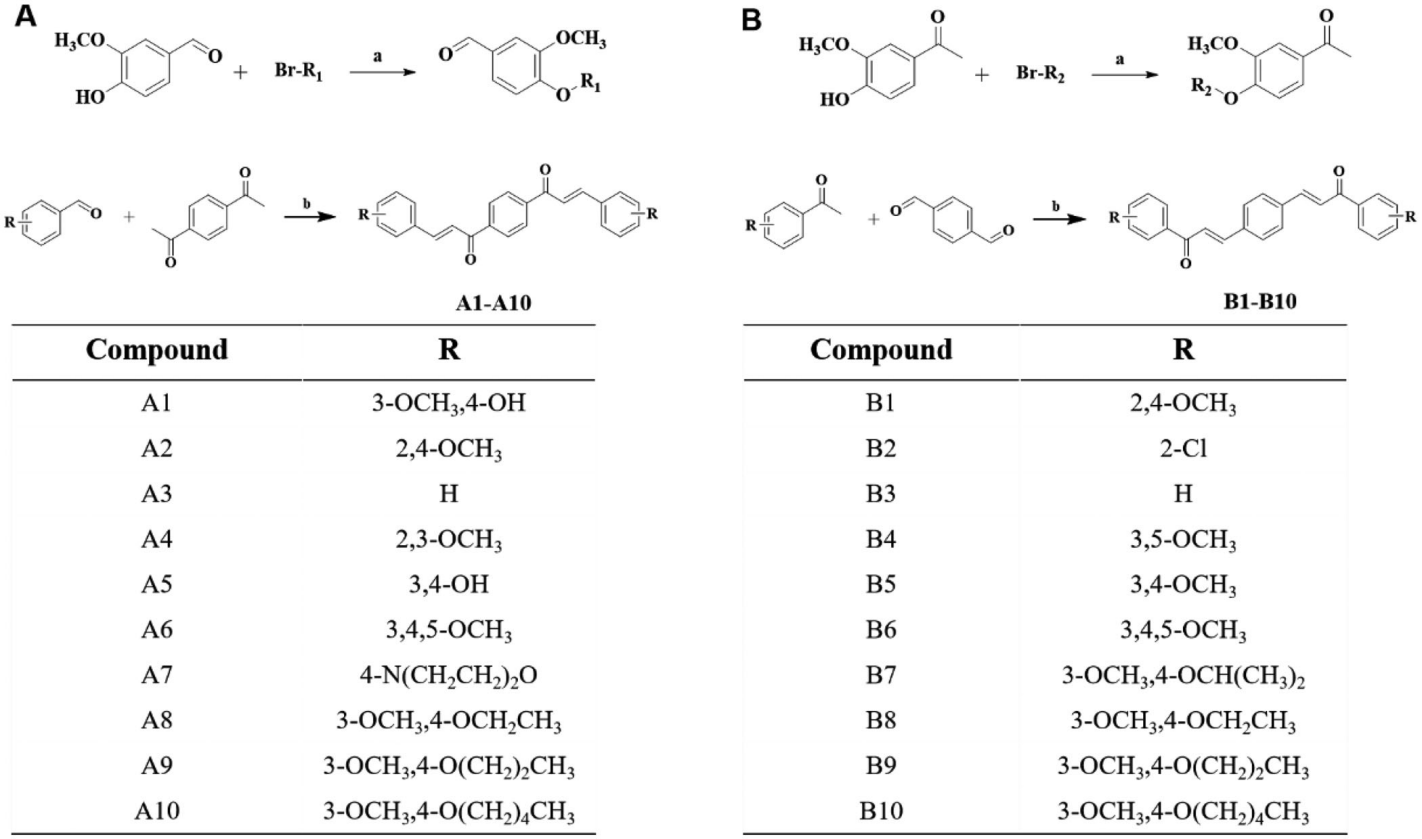
Chemical synthesis. (A) General synthesis steps and compound structure of dicarbonyl curcumin analogues A. (B) General synthesis steps and compound structure of dicarbonyl curcumin analogues B. Reagents and conditions: (a) DMF, K_2_CO_3_, 80 °C; (b) EtOH, HCl, 78 °C/EtOH, NaOH, rt.

The dicarbonyl curcumin analogues A and B were obtained by aldol condensation reaction of aldehydes and ketones. The substituents of the compounds A and B were shown in Figures 2(A,B). The class A compounds (A1–A10) were synthesised based on 1,4-diacetylbenzene and various substituted benzaldehydes in ethanol solution, and NaOH was used as a catalyst. Among them, the substrate benzaldehyde of compounds A8–A10 were prepared by using 3-OCH_3_, 4-OH benzaldehyde as raw material, and replacing the 4-OH with a saturated halogenated hydrocarbon in K_2_CO_3_/DMF reaction system. The B-type compounds (B1–B10) were synthesised by terephthalaldehyde and different substituted acetophenones. B7–B10 were obtained with a substituted saturated halogenated hydrocarbon which was same as class A (Figure 2(A,B)). The synthesis method was simple and easy to operate. The yield of all the final compounds was above 50%. All final novel or unreported compounds are >95% pure by HPLC analysis.

### 2.3. Anti-tumour activity screening and QSAR model constructing

The anti-tumour activity of above 20 compounds was detected in H460 cells and A549 cells by MTT assay (Table 1). The results showed that some compounds exhibited better inhibitory activity than curcumin against both two cancer cells, the activity such as A1, B2, B7 were increased nearly threefold. In general, the inhibitory activity of B-series compounds on two tumour cells was better than that of A-series compounds, and the inhibitory activity on H460 cells was better than that of A549 cells. Among them, B2 was particularly prominent, the IC_50_ for H460 cells and A549 cells were 2.3 μM and 9.6 μM, respectively.

**Table 1.** Cytotoxicity of compounds on human lung cancer cells.

Compound	IC_50_^a^ (μM) for cell lines	
	H460	A549
A1	6.4 ± 1.5	11.2 ± 8.1
A2	>60^b^	>60^b^
A3	7.4 ± 1.9	>60^b^
A4	14.7 ± 2.1	19.9 ± 5.4
A5	14.8 ± 3.4	56.5 ± 18.0
A6	>60^b^	>60^b^
A7	>60^b^	23.0 ± 3.9
A8	>60^b^	>60^b^
A9	>60^b^	>60^b^
A10	>60^b^	>60^b^
B1	>60^b^	19.9 ± 1.6
B2	2.3 ± 0.5	9.6 ± 0.6
B3	>60^b^	>60^b^
B4	10.3 ± 4.0	>60^b^
B5	40.6 ± 17.5	>60^b^
B6	>60^b^	52.1 ± 23.2
B7	7.5 ± 3.7	12.4 ± 2.5
B8	23.3 ± 2.1	33.4 ± 15.8
B9	>60^b^	>60^b^
B10	>60^b^	>60^b^
Curcumin	35.0 ± 4.5	26.6 ± 1.4

^a^IC_50_ was the concentration of a drug that reduced cell viability by 50% relative to the untreated control.

^b^The maximum use of the compound concentration.

**Figure 3.**
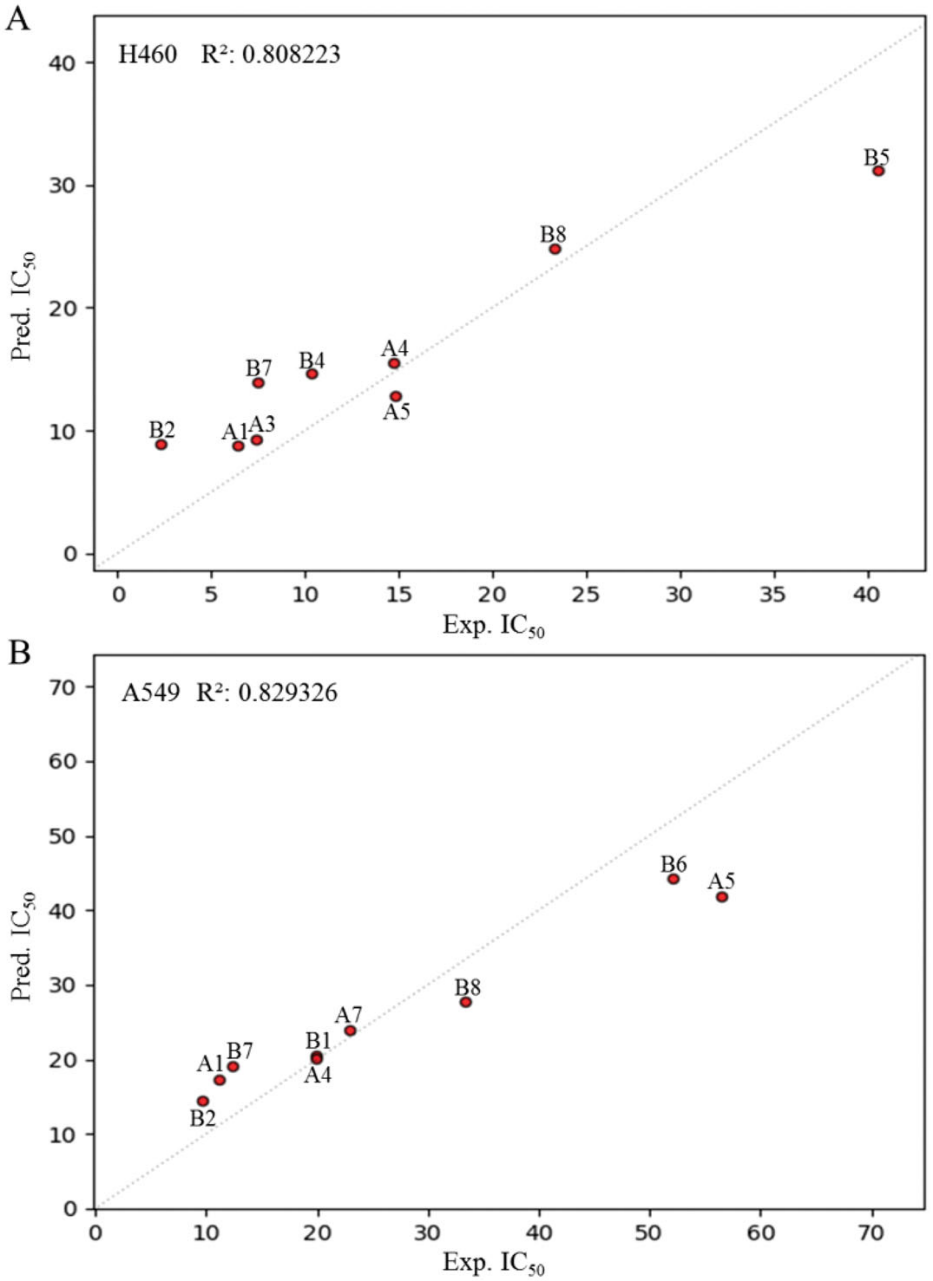
The construction of QSAR model. (A,B) The molecular descriptors of compounds based on physicochemical properties and molecular structure were calculated. The QSAR model was constructed by using RF algorithm.

To further demonstrate the SAR of these compounds in their inhibitory activity, a quantitative SAR (QSAR) was calculated for some compounds with relatively great activity^20^. QSAR based on artificial intelligence (AI) and machine learning has been used, and random forest (RF) algorithm is one of the effective AI algorithms. It could be seen in Figure 3 that an excellent QSAR model was obtained with the RF, which exhibited a high squared regression coefficient (*R*^2^ = 0.808223 for H460 cells, *R*^2^ = 0.829326 for A549 cells). However, it still required more data to improve the model in the follow-up study. Taken together, the QSAR results on the inhibitory activities of the dicarbonyl curcumin analogues may provide valuable information for the further design of novel anti-tumour agents.

### 2.4. The stability and *in vitro* anti-tumour effect of B2

**Figure 4.**
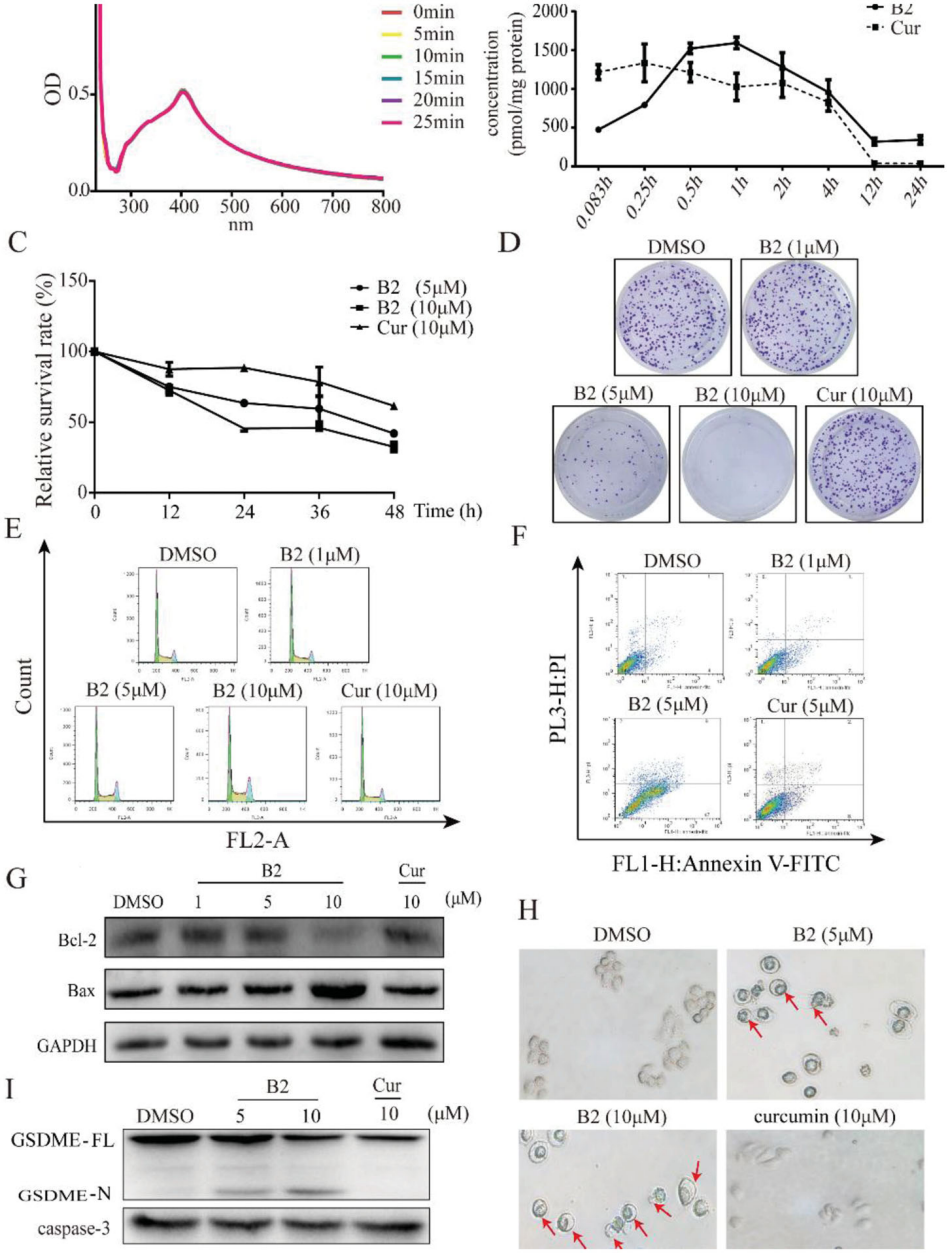
The stability and anti-tumour activity *in vitro*. (A) The absorption spectrum of B2 at different times was detected by ultraviolet spectra assay. (B) The change in concentrations of B2 and curcumin in H460 cells. (C) The cell survival rate was detected by MTT assay after 12, 24, 36, and 48 h treatment of B2 and curcumin. (D) Clonogenic assay of H460 cells treated with B2 and curcumin as indicated. (E) H460 cells were treated with increasing concentrations of B2 and curcumin. The effect of B2 and curcumin in cell cycle was detected using flow cytometer. (F) The cell apoptosis was detected following the treatment of B2 and curcumin. (G) Western blot was performed to detect the effect of B2 on bcl-2 protein after 20 h treatment. (H) The changes in cell morphology were detected after 28 h of B2 treatment. (I) Western blot was performed to detect the effect of B2 on the pyroptosis-related proteins.

To test whether the preferred compound B2 also exhibited great stability and improved the shortcoming of curcumin, the ultraviolet spectra assay was carried out. It was observed that the absorption intensity of B2 in phosphate buffer barely changed (Figure 4(A)), which suggested B2 had relatively great stability. In addition, we used a model of cellular metabolism to examine the change in concentration of B2 in H460 cells. Results showed that curcumin peaked at 15 min, with 50% curcumin metabolised at 4 h and after 12 h almost none, while B2 concentration in the cell was a slow-rising process, reaching its peak in about an hour and a fraction of B2 could be detected in the cell after 12 h (Figure 4(B)). These results indicated that B2 was more stable than curcumin. Besides, it was found that treatment of B2 could significantly inhibit the growth of H460 cells in a dose-dependent and time-dependent manner (Figure 4(C,D)), while curcumin had much lower inhibition rate at the same concentrations. It was maybe the character of slower degradation and accelerated uptake of B2 *in vitro*, contributing to its anti-tumour effect. To elucidate the related mechanism by which to inhibit cell growth, the following work was carried out. Mass of study had reported that curcumin and its analogues could induce cell cycle arrest in various cancer cells^14^^,^^18^, the results showed that B2 also arrested G2/M phase in a dose-dependent manner (Figure 4(E)). Further, it is increasingly being recognised that chemotherapy drugs would induce cell apoptosis and pyroptosis at different stages, and thus exerted its anti-tumour effects^21^. It was observed that treatment with B2 for 20 h resulted in cell apoptosis (Figure 4(F)). In addition, B2 inhibited the expression of Bcl-2 and promoted the expression of Bax that leading to apoptosis (Figure 4(G))^22^. Through further observation, the application of B2 eventually resulted in cell lysis that is the appearance of pyroptosis (Figure 4(H)), while it had no obvious effect under the same concentration of curcumin. It has been reported that caspase-3 was no longer a unique hallmark of apoptosis, but also a hallmark of pyroptosis^21^. Under the action of caspase-3, the pyroptosis-related protein GSDME could be cleaved and generated a GSDME-N fragment^21^. It was observed that B2 could decrease the expression of caspase-3 in a concentration-dependent manner and then induced the generation of GSDME-N (Figure 4(I)). Taken together, it was clear that B2 exhibited anti-tumour activity via the switch from cell apoptosis to pyroptosis.

### 2.5. B2 induced pyroptosis by up-regulating ROS in cancer cells

**Figure 5.**
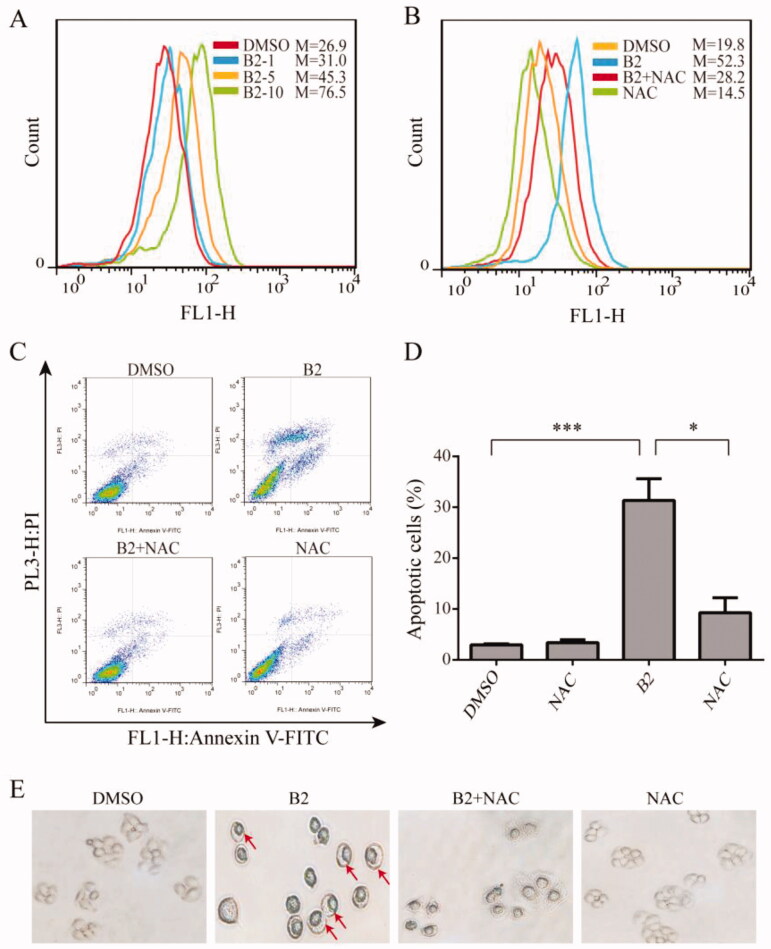
B2 induced H460 cell apoptosis and pyroptosis through ROS up-regulation. (A) The generation of ROS was measured following increasing concentrations of B2 treatment. (B) H460 cells were pre-treated with NAC for 1 h before exposure to B2 (10 μM) for 9 h. The ROS generation was detected using flow cytometer. (C) Following the pre-treatment of H460 cells and NAC for 1 h, flow cytometer were performed to detect the reversal effect of NAC on cell apoptosis. (D) The proportion of cell apoptosis from (B) was calculated. *P < 0.05 versus B2 group; ***P < 0.001 versus control group. (E) H460 cells were treated with B2 for 28 h following pre-treatment of NAC for 1 h. The reversal effect of NAC on cell pyroptosis was detected.

In general, many types of cancer cells maintain high ROS levels due to increased ROS production and decreased ROS scavenging capacity^23^. Therefore, cancer cells are more susceptible to ROS-induced damage by exogenous factors, which means manipulating ROS levels through redox regulation can selectively kill cancer cells without causing significant toxicity to normal cells^24^. Curcumin also increases ROS levels in a variety of cancer cells^25^. Therefore, we examined the effect of B2 on ROS levels in H460 cells. B2 treatment induced a dose-dependent increase of intracellular ROS level for 9 h, and this effect could be reversed by ROS inhibitor NAC (Figure 5(A,B)). To verify that B2 induced cell apoptosis was associated with ROS up-regulation, flow cytometry was performed. Compared to B2 group, the percentage of apoptotic cells pre-incubated with NAC was significantly reduced (Figure 5(C,D)). Similarly, pyroptosis induced by B2 could be effectively reversed by NAC pre-treatment (Figure 5(E)), indicating that the anti-tumour effect of B2 was at least partially achieved by increasing intracellular ROS levels.

### 2.6. B2 resulted in pyroptosis by ROS/ER stress pathways in cancer cells

**Figure 6.**
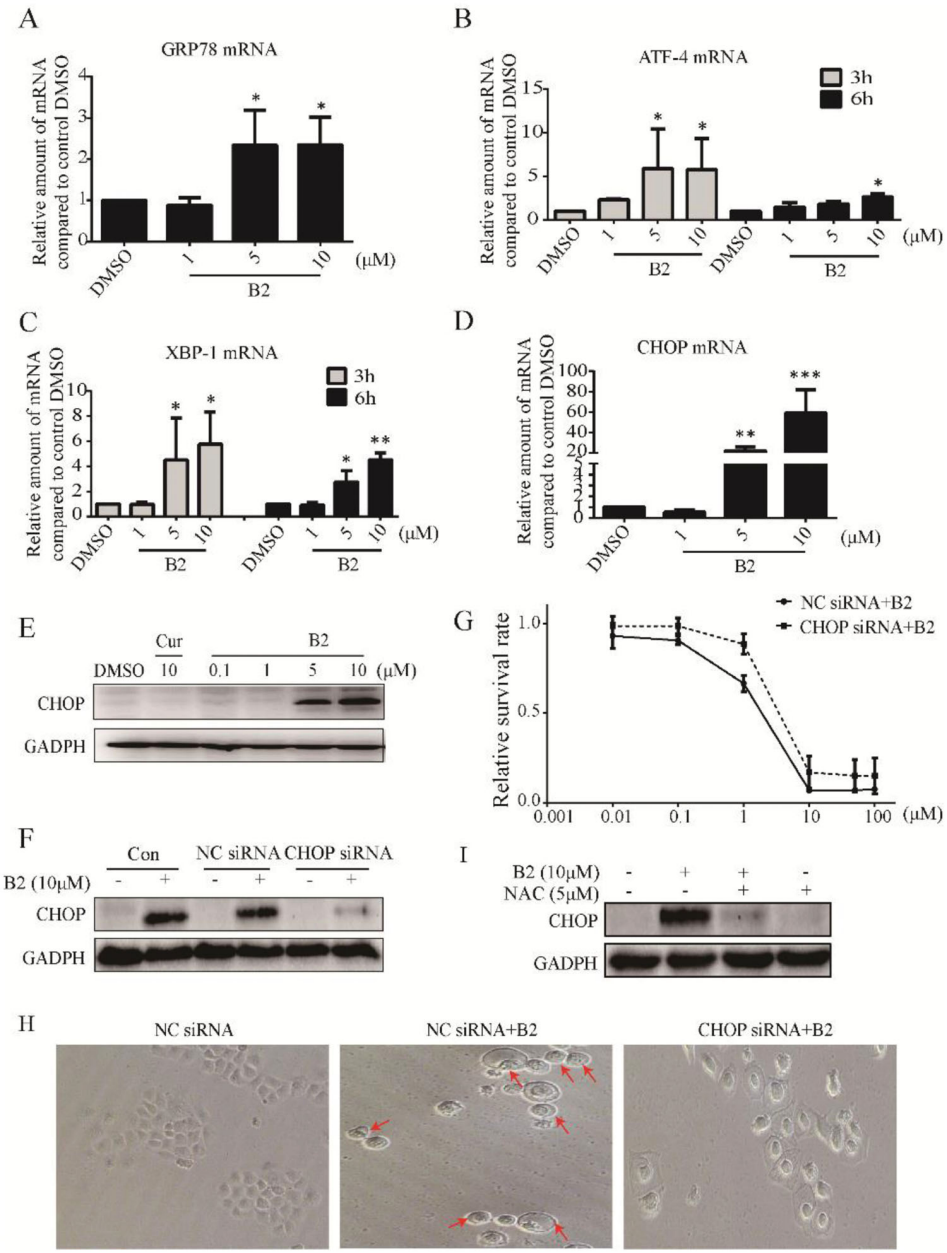
5B induced cell apoptosis and pyroptosis was dependent on the ROS mediated ER stress activation. (A–D) H460 cells were treated with different concentrations of B2 for various time periods. The mRNA levels of GRP78, ATF-4, XBP-1 and CHOP were detected using iQ5 Multi-Colour Real-Time PCR Assay Kit. *P < 0.05 versus control group; **P < 0.01 versus control group; ***P < 0.001 versus control group. (E) Western blot was performed to detect the effect of B2 and curcumin on the expression of CHOP after 12 h treatment. (F) H460 cells were pre-transfected with CHOP-siRNA or negative sequence before exposure to B2 for 12 h. The expression of protein was detected by western blot. (G) The effect of B2 on the cell survival rate in the CHOP-silenced cells and negative sequence transfected cells. The viability of cells was measured using MTT assay. (H) The cells that had undergone RNA interference were treated with B2 for 28 h. The changes in cell morphology were observed through inverted microscope. (I) The effect of NAC on CHOP expression. H460 cells were pre-treated with NAC for 1 h before exposure to B2 for 12 h. Western blot was used to detect the change in CHOP expression.

The ER has many functions in normal cellular, especially in folding and post-translational modification of secretory proteins and membrane proteins. Various physiopathological conditions might break ER homeostasis and lead to unfolded protein response (UPR)^26^. When cells undergo persistent or non-recoverable stimuli, such as high concentrations of ROS, it will activate the ER stress-mediated cell death pathway^27^^,^^28^. Therefore, we speculated that B2 mediated H460 cell death might through the pathway of ER stress. In the initial phase of ER stress, cells will increase the expression of Glucose-regulated protein 78 (GRP78) and to induce ER stress^29^. Assuredly, B2 dose-dependently increased the mRNA level of GRP78 in H460 cells (Figure 6(A)). The mRNA levels of the downstream transcription factor X-box binding proteins-1 (XBP-1) and activating transcription factor 4 (ATF-4) were then determined by RT-PCR. Incubation with B2 for 6 h significantly increased the mRNA expression of ATF-4 and XBP-1 in a dose-dependent manner, and ATF-4 and XBP-1 as transcription factors entered the nucleus to regulate ER stress (Figure 6(B,C))^30^^,^^31^. Besides, many other proteins play an important role in ER stress pathway likewise, and CHOP protein is the most important protein in ER stress-mediated cell death^32^. B2 significantly increased the mRNA level and protein expression of CHOP at concentrations >5 μM, and this effect was reversed by NAC, whereas curcumin with the concentration of 10 μM had no effect on the protein expression of CHOP (Figure 6(D,E,I)). To confirm the mechanism of B2, we found that after 12 h of B2 incubation, the expression of CHOP in CHOP-silenced transfected H460 cell line was significantly decreased compared to negative cell line transfected with empty vector (Figure 6(F)). Moreover, it was easy to find that in CHOP-silenced transfected cells, the apoptosis and pyroptosis induced by B2 were significantly restricted in relative to negative cells (Figure 6(G,H)). In conclusion, the above experiments indicated that the inhibitory effect of B2 on tumour cells was achieved through ROS-activated ER stress-mediated cell death pathway.

### 2.7. Anti-tumour activity *in vivo* of B2

**Figure 7.**
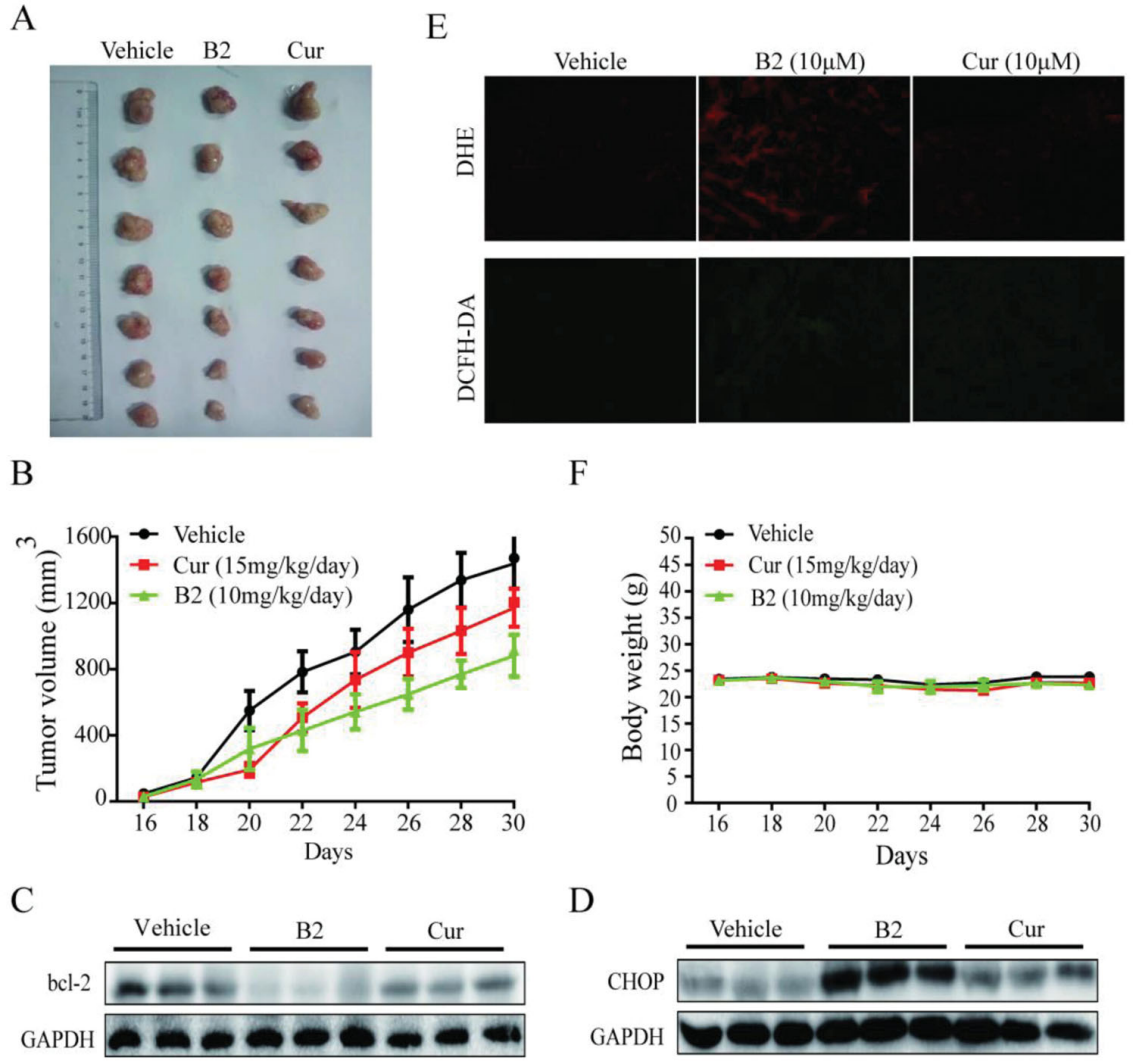
Anti-tumour activity *in vivo* of B2. (A) The tumours were excised and photographed on the day the mice were sacrificed. (B) Tumour volume was monitored. (C,D) The expression of bcl-2 and CHOP in tumour tissues. (E) Frozen sections were stained with DHE probes and DCFH-DA probes, respectively. The changes in fluorochromes were analysed by fluorescence microscope. (F) Body weight was measured once every two days.

The anti-tumour activity of compound B2 *in vivo* was further studied by nude mice model. Once the tumour volumes could be measured, curcumin (15 mg/kg/day) and B2 (10 mg/kg/day) were then administered for 15 days. Compared with the blank group, the tumour volume in the B2 group was significantly different (*P* < 0.05), but there was no significant difference in the curcumin group (*P* > 0.05). This shows that B2 treatment effectively inhibited the tumour growth, which was a significant improvement over curcumin (Figure 7(A,B)). What is more, B2 had no effect on the weight of nude mice (Figure 7(F)). Then, the expression of CHOP and bcl-2 proteins in three groups were tested by western blot. B2 could up-regulate CHOP expression and decrease bcl-2 expression, which was obviously better than curcumin (Figure 7(C,D)). DHE and DCFH-DA were then used to measure superoxide anion and hydrogen peroxide, respectively. DHE could be oxidised by intracellular superoxide and bound to DNA to form ethidium (ETH)^33^. DCFH-DA was cleaved by esterase to DCFH and then oxidised by hydrogen peroxide to form DCF^33^. The fluorescence intensity of ETH and DCF in tumour tissues of B2 group was significantly stronger than that of curcumin group (Figure 7(E)), indicating that B2 also could induce the generation of ROS *in vivo*, which was consistent with the result *in vitro*. These results suggested that B2 possessed the capacity of inhibiting the growth of tumours *in vivo*, and that may via the ROS-activated ER stress-mediated cell death pathway.

## 3. Materials and methods

### 3.1. Synthesis

#### 3.1.1. Chemistry

Reagents and solvents for the synthesis were commercially available and obtained from Sigma-Aldrich (St Louis, Missouri, USA) and Aladdin (Shanghai, China), which were used without further purification. Silica gel (GF254) for column chromatography (200–300 mesh) was obtained from Aladdin. Melting points were measured on a Fisher-Johns melting apparatus and were uncorrected. Mass spectra (MS) were recorded on an Agilent 1100 LC-MS (Agilent, Palo Alto, CA, USA). The ^1^H-NMR spectra data were recorded on a 600 MHz spectrometer (Bruker Corporation, Switzerland) with TMS as an internal standard. The purity of the compounds was measured by HPLC (Shimadzu LC-20AD, Japan; 80% methanol: 20% water for elution).

The published structures only list the colour, yield, melting range, MS^19^^,^^33–35^. The spectral data of final unreported compounds are listed as the following:

(2E,2′E)-1,1′-(1,4-phenylene)bis(3-(4-hydroxy-3-methoxyphenyl)prop-2-en-1-one) (A1): yellow powder, 68.8% yield, mp 222.0 °C–225.6 °C. LC-MS *m/z*: 431.21 (M + H)^+^, calcd for C_26_H_22_O_6_: 430.14.

(2E,2′E)-1,1′-(1,4-phenylene)bis(3-(2,4-dimethoxyphenyl)prop-2-en-1-one) (A2): light yellow powder, 59.8% yield, mp 201.0 °C–202.5 °C. LC-MS *m/z*: 459.13 (M + H)^+^, calcd for C_28_H_26_O_6_: 458.17.

(2E,2′E)-1,1′-(1,4-phenylene)bis(3-phenylprop-2-en-1-one) (A3): light yellow powder, 59.8% yield, mp 191.3 °C–198.3 °C. LC-MS *m/z*: 338.28 (M + H)^+^, calcd for C_24_H_18_O_2_: 338.13.

(2E,2′E)-1,1′-(1,4-phenylene)bis(3-(2,3-dimethoxyphenyl)prop-2-en-1-one) (A4): light yellow powder, 60.4% yield, mp 192.8 °C–194.6 °C. ^1^H-NMR (600 MHz, CDCl_3_), δ: 8.167 (d, *J*  =  16.2 Hz, 2H, H-β × 2), 8.143 (s, 4H, Ar-H × 4), 7.738 (d, *J*  =  8.4 Hz, 2H, H-6 × 2), 7.518 (d, *J*  =  1.8 Hz, 2H, H-4 × 2), 7.494 (d, *J*  =  16.2 Hz, 2H, H-α × 2), 7.354 (dd, *J*_1_ = 1.8 Hz, *J*_2_ = 8.4 Hz, 2H, H-5 × 2), 1.587 (s, 12H, 2-OCH_3_×2, 3-OCH_3_×2). ^13^C NMR (101 MHz, CDCl_3_) δ: 190.44, 153.28, 149.13, 141.37, 140.66, 128.81, 124.30, 123.42, 119.71, 114.55, 61.41, 55.96. LC-MS *m/z*: 459.20 (M + H)^+^, calcd for C_28_H_26_O_6_: 458.17.

(2E,2′E)-1,1′-(1,4-phenylene)bis(3-(3,4-dihydroxyphenyl)prop-2-en-1-one) (A5): yellow powder, 78.3% yield, mp 300+°C. *R_t_*: 3.498 min; purity: 97.496%. ^1^H-NMR (600 MHz, DMSO-d_6_), δ: 8.217 (t, *J*  =  19.2 Hz, 4H, Ar-H × 4), 8.098 (d, *J*  =  8.4 Hz, 2H, H-β × 2), 7.641 (t, *J*  =  15.0 Hz, 4H, H-2 × 2, H-α × 2), 7.233–7.205 (m, 2H, H-5 × 2), 6.836–6.816 (m, 2H, H-6 × 2). ^13^C NMR (101 MHz, DMSO-d_6_) δ: 198.29, 189.19, 149.53, 146.79–145.86, 141.84, 141.46, 139.96, 129.39–128.62, 126.64, 123.04, 118.91, 116.20, 27.54. LC-MS *m/z*: 403.15 (M + H)^+^, calcd for C_24_H_18_O_6_: 402.11.

(2E,2′E)-1,1′-(1,4-phenylene)bis(3-(3,4,5-trimethoxyphenyl)prop-2-en-1-one) (A6): yellow powder, 78.3% yield, mp 223.8 °C–224.6 °C. LC-MS *m/z*: 519.23 (M + H)^+^, calcd for C_30_H_30_O_8_: 518.19.

(2E,2′E)-1,1′-(1,4-phenylene)bis(3-(4-morpholinophenyl)prop-2-en-1-one) (A7): yellow powder, 78.3% yield, mp 272.1 °C–274.2 °C. LC-MS *m/z*: 509.18 (M + H)^+^, calcd for C_32_H_32_N_2_O_4_: 508.24.

(2E,2′E)-1,1′-(1,4-phenylene)bis(3-(4-ethoxy-3-methoxyphenyl)prop-2-en-1-one) (A8): aint yellow powder, 67.5% yield, mp 169.7–170.8 °C. *R_t_*: 2.23 min; purity: 100.00%. ^1^H-NMR (600 MHz, CDCl_3_), δ: 8.125 (s, 4H, Ar-H × 4), 7.805 (d, *J*  =  15.6 Hz, 2H, H-β × 2), 7.401 (d, *J*  =  15.6 Hz, 2H, H-α × 2), 7.258 (dd, *J*_1_ = 1.5 Hz, *J*_2_ = 8.1 Hz, 2H, H-6 × 2), 7.197 (d, *J*  =  1.2 Hz, 2H, H-2 × 2), 6.939 (d, *J*  =  8.4 Hz, 2H, H-5 × 2), 4.208–4.173 (m, 4H, 4-OCH_2_×2), 3.976 (s, 6H, 3-OCH_3_×2), 1.527 (t, *J*  =  6.6 Hz, 6H, CH_3_×2). ^13^C NMR (101 MHz, CDCl_3_) δ: 190.25, 151.24, 149.56, 146.12, 141.54, 128.63, 127.50, 123.51, 119.82, 112.29, 110.56, 64.51, 56.12, 14.74. LC-MS *m/z*: 487.17 (M + H)^+^, calcd for C_30_H_30_O_6_: 486.20.

(2E,2′E)-1,1′-(1,4-phenylene)bis(3-(3-methoxy-4-propoxyphenyl)prop-2-en-1-one) (A9): faint yellow powder, 58.9% yield, mp 139.8 °C–140.4 °C. *R_t_*: 2.836 min; purity: 100.00%. ^1^H-NMR (600 MHz, CDCl_3_), δ: 8.126 (s, 4H, Ar-H × 4), 7.805 (d, *J*  =  15.6 Hz, 2H, H-β × 2), 7.399 (d, *J*  =  15.6 Hz, 2H, H-α × 2), 7.251 (d, *J*  =  7.8 Hz, 2H, H-6 × 2), 7.197 (s, 2H, H-2 × 2), 6.931 (d, *J*  =  8.4 Hz, 2H, H-5 × 2), 4.066 (t, *J*  =  6.6 Hz, 4H, 4-OCH_2_×2), 3.969 (s, 6H, 3-OCH_3_×2), 1.956–1.857 (m, 4H, CH_2_×2), 1.085 (t, *J*  =  7.2 Hz, 6H, CH_3_×2). ^13^C NMR (101 MHz, CDCl_3_) δ: 190.29, 151.50, 149.65, 146.15, 141.53, 128.61, 127.44, 123.51, 119.78, 112.47, 110.76, 70.54, 56.18, 22.40, 10.43. LC-MS *m/z*: 515.38 (M + H)^+^, calcd for C_32_H_34_O_6_: 514.24.

(2E,2′E)-1,1′-(1,4-phenylene)bis(3-(3-methoxy-4-(pentyloxy)phenyl)prop-2-en-1-one) (A10): yellow powder, 49.4% yield, mp 136.4 °C–137.5 °C. *R_t_*: 2.851 min; purity: 99.224%. ^1^H-NMR (600 MHz, CDCl_3_), δ: 8.104 (s, 4H, Ar-H × 4), 7.783 (d, *J*  =  15.6 Hz, 2H, H-β × 2), 7.377 (d, *J*  =  15.6 Hz, 2H, H-α × 2), 7.234 (d, *J*  =  6.6 Hz, 2H, H-2 × 2), 7.175 (d, *J*  =  1.8 Hz, 2H, H-6 × 2), 6.907 (d, *J*  =  8.4 Hz, 2H, H-5 × 2), 4.074 (t, *J*  =  6.6 Hz, 4H, 4-OCH_2_×2), 3.946 (s, 6H, 3-OCH_3_×2), 1.907–1.859 (m, 4H, CH_2_×2), 1.474–1.395 (m, 8H, -CH_2_CH_2_×2), 0.941 (t, *J*  =  14.4 Hz, 6H, CH_3_×2). ^13^C NMR (101 MHz, CDCl_3_) δ: 190.27, 151.51, 149.64, 146.14, 141.53, 128.60, 127.42, 123.51, 119.76, 112.42, 110.72, 69.09, 56.17, 28.77, 28.09, 22.48, 14.03. LC-MS *m/z*: 571.23 (M + H)^+^, calcd for C_36_H_42_O_6_: 570.30.

(2E,2′E)-3,3′-(1,4-phenylene)bis(1-(2,4-dimethoxyphenyl)prop-2-en-1-one) (B1): light yellow powder, 56.9% yield, mp 300+ °C. LC-MS *m/z*: 459.26 (M + H)^+^, calcd for C_28_H_26_O_6_: 458.17.

(2E,2′E)-3,3′-(1,4-phenylene)bis(1-(2-chlorophenyl)prop-2-en-1-one) (B2): light yellow powder, 56.9% yield, mp 149.5 °C–150.9 °C. *R_t_*: 9839 min; purity: 96.061%. LC-MS *m/z*: 407.00 (M + H)^+^, calcd for C_24_H_16_Cl_2_O_2_: 406.05.

(2E,2′E)-3,3′-(1,4-phenylene)bis(1-phenylprop-2-en-1-one) (B3): light yellow powder, 56.9% yield, mp 190.5 °C–191.9 °C. LC-MS m/s: 338.55 (M + H)^+^, calcd for C_24_H_18_O_2_: 338.1.

(2E,2′E)-3,3′-(1,4-phenylene)bis(1-(3,5-dimethoxyphenyl)prop-2-en-1-one) (B4): light yellow powder, 56.9% yield, mp 180.1 °C–182.3 °C. LC-MS *m/z*: 459.26 (M + H)^+^, calcd for C_28_H_26_O_6_: 458.17.

(2E,2′E)-3,3′-(1,4-phenylene)bis(1-(3,4-dimethoxyphenyl)prop-2-en-1-one) (B5): light yellow powder, 56.9% yield, mp 213.4 °C–215.6 °C. LC-MS *m/z*: 459.20 (M + H)^+^, calcd for C_28_H_26_O_6_: 458.17.

(2E,2′E)-3,3′-(1,4-phenylene)bis(1-(4-hydroxy-3-methoxyphenyl)prop-2-en-1-one) (B6): brown powder, 70.2% yield, mp 195.5 °C–196.9 °C. LC-MS *m/z*: 519.16 (M + H)^+^, calcd for C_30_H_30_O_8_: 518.19.

(2E,2′E)-3,3′-(1,4-phenylene)bis(1-(4-isopropoxy-3-methoxyphenyl)prop-2-en-1-one) (B7): faint yellow powder, 53.8% yield, mp 197.9 °C–198.5 °C. *R_t_*: 2.839 min; purity: 95.724%. ^1^H-NMR (600 MHz, CDCl_3_), δ: 7.810 (d, *J*  =  15.6 Hz, 2H, H-α × 2), 7.696 (s, 4H, Ar-H × 4), 7.673 (dd, *J*_1_ = 1.8 Hz, *J*_2_ = 8.4 Hz, 2H, H-6 × 2), 7.638 (d, *J*  =  1.8 Hz, 2H, H-2 × 2), 7.611 (d, *J*  =  15.6 Hz, 2H, H-β × 2), 6.949 (d, *J*  =  8.4 Hz, 2H, H-5 × 2), 4.721–4.680 (m, 2H, 4-OCH × 2), 3.957 (s, 6H, 3-OCH_3_×2), 1.441 (d, *J*  =  6.0 Hz, 12H, CH_3_×4). ^13^C NMR (101 MHz, CDCl_3_) δ: 188.31, 152.09, 150.23, 142.66, 136.96, 130.94, 128.88, 123.03, 122.69, 112.86, 111.50, 71.34, 56.18, 21.98. LC-MS *m/z*: 515.17 (M + H)^+^, calcd for C_32_H_34_O_6_: 514.24.

(2E,2′E)-3,3′-(1,4-phenylene)bis(1-(4-ethoxy-3-methoxyphenyl)prop-2-en-1-one) (B8): faint yellow powder, 66.0% yield, mp 193.0 °C–193.9 °C. *R_t_*: 2.832 min; purity: 95.439%. ^1^H-NMR (600 MHz, CDCl_3_), δ: 7.809 (d, *J*  =  15.6 Hz, 2H, H-α × 2), 7.684 (d, *J*  =  12.0 Hz, 6H, Ar-H × 4, H-6 × 2), 7.637 (s, 2H, H-2 × 2), 7.609 (d, *J*  =  15.6 Hz, 2H, H-β × 2), 6.935 (d, *J*  =  8.4 Hz, 2H, H-5 × 2), 4.223–4.189 (m, 4H, 4-OCH_2_×2), 3.975 (s, 6H, 3-OCH_3_×2), 1.525 (t, *J*  =  7.2 Hz, 6H, CH_3_×2). ^13^C NMR (101 MHz, CDCl_3_) δ: 188.32, 152.92, 149.51, 142.70, 136.96, 131.00, 128.88, 123.13, 122.66, 111.01, 64.57, 56.14, 14.67. LC-MS *m/z*: 487.17 (M + H)^+^, calcd for C_30_H_30_O_6_: 486.20.

(2E,2′E)-3,3′-(1,4-phenylene)bis(1-(3-methoxy-4-propoxyphenyl)prop-2-en-1-one) (B9): faint yellow powder, 59.2% yield, mp 151.9 °C–153.8 °C. *R_t_*: 2.836 min; purity: 100.00%. ^1^H-NMR (600 MHz, CDCl_3_), δ: 7.808 (d, *J*  =  15.6 Hz, 2H, H-α × 2), 7.695 (s, 4H, Ar-H × 4), 7.680 (d, *J*  =  7.2 Hz, 2H, H-6 × 2), 7.636 (s, 2H, H-2 × 2), 7.609 (d, *J*  =  15.6 Hz, 2H, H-β × 2), 6.935 (d, *J*  =  8.4 Hz, 2H, H-5 × 2), 4.079 (t, *J*  =  7.2 Hz, 4H, 4-OCH_2_×2), 3.967 (s, 6H, 3-OCH_3_×2), 1.934–1.895 (m, 4H, CH_2_×2), 1.075 (t, *J*  =  7.8 Hz, 6H, CH_3_×2). ^13^C NMR (101 MHz, CDCl_3_) δ: 188.30, 153.18, 149.60, 142.66, 136.95, 130.96, 128.88, 123.14, 122.66, 111.18, 70.56, 56.18, 22.37, 10.43. LC-MS *m/z*: 515.31 (M + H)^+^, calcd for C_32_H_34_O_6_: 514.24.

(2E,2′E)-3,3′-(1,4-phenylene)bis(1-(3-methoxy-4-(pentyloxy)phenyl)prop-2-en-1-one) (B10): faint yellow powder, 50.4% yield, mp 142.0 °C–143.2 °C. *R_t_*: 2.846 min; purity: 100.00%. ^1^H-NMR (600 MHz, CDCl_3_), δ: 7.812 (d, *J*  =  15.6 Hz, 2H, H-α × 2), 7.699 (s, 4H, Ar-H × 4), 7.681 (dd, *J*_1_ = 1.8 Hz, *J*_2_ = 8.4 Hz, 2H, H-6 × 2), 7.635 (d, *J*  =  1.8 Hz, 2H, H-2 × 2), 7.612 (d, *J*  =  15.6 Hz, 2H, H-β × 2), 6.936 (d, *J*  =  7.2 Hz, 2H, H-5 × 2), 4.112 (t, *J*  =  7.2 Hz, 4H, 4-OCH_2_×2), 3.969 (s, 6H, 3-OCH_3_×3), 1.930–1.882 (m, 4H, CH_2_×2), 1.488–1.394 (m, 8H, CH_2_CH_2_×2), 0.948 (t, *J*  =  7.2 Hz, 6H, CH_3_×2). ^13^C NMR (101 MHz, CDCl_3_) δ:188.32, 153.20, 149.60, 142.67, 136.96, 130.95, 128.88, 123.14, 122.68, 111.15, 69.14, 56.19, 28.73, 28.09, 22.48, 14.02. LC-MS *m/z*: 571.51 (M + H)^+^, calcd for C_36_H_42_O_6_: 570.30.

#### 3.1.2. Synthetic procedures

Synthesis of class A compounds: when the benzaldehyde had a hydroxyl group, it was dissolved in absolute ethanol (15–50 ml), and then 2.5 mM of 1,4-diacetylbenzene and 3–5 drops of concentrated sulphuric acid were added thereto, followed by an oil bath at 78 °C while other benzaldehyde at room temperature can be catalysed by 40% NaOH. The sodium hydroxide used in this experiment as a catalyst and the progress of the reaction was monitored by TLC. Once the reaction has completed, the water was added into the not treated or concentrated reaction solution for 0.5 to 72 h at 0 °C to 4 °C. The precipitate was then filtrated, washed with 10% aqueous ethanol, and purified by column chromatography using petroleum ether and ethyl acetate as mobile phases.

Synthesis of A8-A10 substrate benzaldehyde: 6 mM of benzaldehyde with 3-OCH_3_, 4-OH was firstly dissolved in 10 ml DMF solution, 6 mM of corresponding halogenated hydrocarbon and 7.2 mM of K_2_CO_3_ (as a catalyst) were then followed. The reaction was carried by magnetic stirring at 80 °C oil bath and the progress was monitored by TLC. After completion of the reaction, the extraction was carried out with a saturated sodium chloride solution and ethyl acetate, and purified by column chromatography using petroleum ether and ethyl acetate as mobile phases.

Synthesis of class B compounds: Following the dissolution of various substituted acetophenones (5 mM) in 15 to 50 ml absolute ethanol, 2.5 mM of 1,4-phthalaldehyde were added into the reaction solution, and other reaction conditions and follow-up treatment methods were such as class A.

Substrate acetophenone synthesis of B7-B10: 6 mM of 3-OCH_3_, 4-OH acetophenone was firstly dissolved in 10 ml DMF solution, and 6 mM of corresponding halogenated hydrocarbon and 7.2 mM of K_2_CO_3_ were followed. Other reaction conditions and follow-up treatment methods were such as class A.

### 3.2. Molecular stability assay

The energy of the chemical bond cleavage at 298 K and 101.325 kPa is equal to the reaction enthalpy A-B(g)→A·(g)+B·(g), which can be defined as the bond dissociation of A-B. As the BDE and bond dissociation enthalpy of many organic compounds are usually approximately equal^36^, the BDE of the material at 298 K and 101.325 kPa that could be calculated according to the formula (1)^37^: BDE(A-B) = E0(A·) + E0(B·) − E0(A-B) (1), E0(A·) and E0(B·) represent the total energy of the radical, E0(A-B) represents the total energy of the molecule. The molecular and radical radicals were optimised at the B3LYP/6-31G(d) level using computational Gaussian software, and the total energy of the six compounds was calculated separately. For the fracture-saturated bond is higher than the energy of rupturing unsaturated bond, the formula (1) thus is chosen to calculate the energy of each saturated bond in the structural interrupt chain. The convergence accuracy of the calculation results is the default value of the program. The vibration analysis shows that all the optimised configurations have no virtual frequencies at calculation level, and are local minimum points on the potential energy surface.

### 3.3. Cell lines

Cell lines H460, A549 were purchased from ATCC (Manassas, VA). Cells were cultured in RPMI 1640 medium (Invitrogen, Carlsbad, CA) containing 10% heat inactivated FBS (Atlanta Biologicals Inc., Lawrenceville, GA), 100 IU/ml penicillin, 100 μg/ml streptomycin, and incubated at 37 °C.

### 3.4. Methyl thiazolyl tetrazolium (MTT) assay

The cells were diluted to 30,000 cells/ml and 100 μl of the diluted cell suspension was added to each well of the 96-well plate. After the cell cultured overnight, the fresh medium was replaced, drug dilutions was then added into each well. The cell viability was detected after 12, 24, 36, 48, and 72 h incubation with tested compounds respectively. All cells were then treated with 25 μl MTT (5 mg/ml) for 4 h. The absorbance was detected following the mixture of DMSO. DMSO as a negative control and curcumin as a positive control.

### 3.5. Construction of QSAR model

The molecular descriptors through the Open Source Toolkit RDKit were first calculated, which was a basic requirement for establishing QSAR model. The calculated results included 208 dimensional 2 D molecular descriptors, such as molecular weight, topological polarity surface area and lipid water partition coefficient. Among them, descriptors with equal contents and no difference were deleted. After that, 99 dimensional molecular descriptors were retained in the model corresponding to H460 cells, and 114 dimensional molecular descriptors were retained in the model corresponding to A549 cells. The screened molecular descriptors were sent to the RF as input, and the QSAR model was obtained by computer training. In this modelling process, the default number of sub decision trees was 800.

### 3.6. Ultraviolet spectra assay

Compounds were dissolved in 1 ml of DMSO to obtain a final concentration of 40 μM. Then, 2 μl of the compound solution and 98 μl of phosphate buffer (pH 7.4) were thoroughly mixed and assayed using the spectrum Max M5 (Molecular Devices, USA) to detect the OD value on 250–800 nm. Absorption curves over 25 min were recorded at 5-min intervals and the temperature measured was 25 °C. In this experiment, the decrease of the absorption spectrum at different times means the degradation of the compound, which reflected the stability of the compound. Curcumin was served as a positive control.

### 3.7. Stability of B2 in H460 cells

Cells were treated with B2 (20 μM) and curcumin for a specific time interval. After that, the cells were washed twice with 2 ml cold PBS and harvested with 100 μl of cell lysis buffer. The B2 and curcumin were extracted with acetonitrile, their concentrations were determined by HPLC and the protein content was determined using a Bio-Rad protein assay reagent. The intracellular concentration of the drug was normalised to pmol/mg protein, Agilent LC (Series 1200) for HPLC analysis, mobile phase consisted of acetonitrile and water (60/40 v/v). Chromatographic separation was achieved using a Beckman C18 reverse phase column at a flow rate of 1 ml/min at room temperature and the B2 and curcumin eluates were monitored at a wavelength of 320 nm.

### 3.8. Cell colony formation assay

H460 cells were seeded in 6-well plates with the cell density of 1000 per hole. The next day, cells were treated with B2 or curcumin. After 3 h incubation with tested compounds, the fresh medium was replaced. Eight days later, cells were fixed with methanol (1%) and formaldehyde (1%) for 15 min, stained with crystal violet for 10 min.

### 3.9. Cell cycle analysis

H460 cells were seed on 60-mm plates overnight, and then treated with B2 and curcumin for 10 h. The cells were centrifuged at 1000 rpm for 5 min. The supernatant was discarded and the isolated cells were washed with cold PBS. After that, the cells were fixed with ice-cold 70% ethanol in PBS for 30 min. Propidium iodide (PI) was then added to the tube at a final concentration of 0.05 mg/ml and incubated in the dark for 15 min. Cell cycle analysis was performed in a FACS Calibur flow cytometer (BD Biosciences, CA).

### 3.10. Cell apoptosis analysis

H460 cells were seeded on 60-mm plates overnight and then treated with B2 and curcumin for 20 h. All cells were collected by the pancreatin without EDTA, washed with pre-chilled PBS and resuspended in binding buffer. Cells were stained with FITC Annexin V and PI at room temperature for 15 min. Apoptosis assays were performed in a FACS Calibur flow cytometer (BD Biosciences, CA).

### 3.11. Determination of cellular levels of ROS

H460 cells (5 × 10^5^/well) were inoculated in the Petri dishes of 3 cm diameter and allowed to be cultured overnight. After the treatment with B2 at the indicated concentrations for 9 h, cells were stained with 10 mM DCFH-DA (Beyotime Biotech, Nantong, China) for 30 min at 37 °C, and then harvested and analysed for fluorescence using a FACS Calibur flow cytometer (BD Biosciences, CA). In some experiments, cells were pre-treated with 5 mM NAC before addition of B2.

### 3.12. RNA isolation and real-time quantitative PCR

Following the B2 treatment, total mRNA was isolated from the cells using the Ambion RNAqueous kit. First-strand cDNA of mRNA was obtained using a high-capacity cDNA archive kit. The mRNA levels of CHOP, XBP-1, ATF-4, and GRP78 were quantified using iQ5 Multi-Colour Real-Time PCR Assay Kit (Bio-Rad, Hercules, CA). Actin mRNA as internal control. Ambion RNAqueous kits were purchased from Applied Biosystems Inc. (Foster City, CA).

### 3.13. Western blot analysis

Cells were lysed and the protein concentration in all samples was determined using the Bradford Protein Assay Kit (Bio-Rad, Hercules, CA). Lysates were then analysed by western blot analysis and immunoreactive bands were visualised using ECL kit (Bio-Rad, Hercules, CA). Anti-CHOP, anti-GAPDH, goat anti-rabbit IgG-HRP, goat anti-mouse IgG-HRP antibodies were purchased from Santa Cruz Biotechnology (Santa Cruz, CA). The antibodies for caspase-3, bcl-2 were obtained from Cell Signalling Technology (Danvers, MA). The antibody for GSDME was obtained from Abcam.

### 3.14. Microscopy imaging of cell pyroptosis

For cell pyroptotic analysis, cells were vaccinated in 6-well plates and treated with B2 or curcumin for 28 h. The changes in cell morphology were detected with inverted fluorescence microscope.

### 3.15. Anti-tumour research *in vivo*

All animal experiments were in line with the Wenzhou Medical University Laboratory Animal Care and use of the policy of animal experiments by the Wenzhou Medical College Animal Policy and Welfare Committee approval. Five weeks old athymic BALB/cA-nu/nu female mice (18–22 g) were purchased for *in vivo* experiments. Animals were re-housed at constant room temperature for 12 h: light/dark for 12 h and fed with a standard rodent diet and water. H460 cells were harvested and injected subcutaneously to the right flank (1 × 10^7^ cells in 150 μl PBS) of the mice. After 15 days, mice were injected with B2 (10 mg/kg/day) or curcumin (15 mg/kg/day) intraperitoneally. Tumour volume (*V*  =  0.5×*L*×*W*^2^) was determined by measuring length (*L*) and width (*W*). After 15 days, the animals were sacrificed under ether anaesthesia and the tumours were harvested.

### 3.16. Immunofluorescence assay

Collected tumour tissue was embedded in OCT Compound at −20 °C. Frozen sections were harvested and stained with DHE probes for half an hour. Another part of frozen sections was stained with DCFH-DA probes for 2 h. Fluorochromes were analysed by fluorescence microscope.

### 3.17. RNA interference

H460 cells were cultured in 6-well plates with the cell density of 3 × 10^5^ per hole overnight. The cells were then transfected with CHOP-siRNA or negative sequence using Transfection Agent lipo2000 (Invitrogen, USA). After 8 h of transfection, the culture medium was replaced and the cells were further incubated for the following research.

### 3.18. Statistical analysis

All experiments were repeated in triplicate (*n*  =  3). Data are presented as mean ± SEM. GraphPad Pro Prism 5.0 (GraphPad, San Diego, CA) was performed for all statistical analysis. Student’s *t*-test was used to analyse the differences between datasets. *P* < 0.05 was considered significant.

## 4. Discussion

Curcumin is prone to be protonized and degraded by external environment due to its β-diketone structure^11^. Related literature indicates that the cleavage of curcumin occurs mainly at different positions of β-diketone methyl bridge, resulting in two different monoalkenyl catabolic products^38^^,^^39^. For instance, the crystallised curcumin obtained by ethanol extraction would produce some by-products such as vanillin (34%), ferulic aldehyde (0.5%), and ferulic acid under 120 h of illumination^40^. The modification of β-diketone by inserting a new structure is one of the effective strategies for changing its BDE and improving the stability of curcumin. The BDE is a physical quantity indicating the strength of a chemical bond and referring to the energy consumed or released by breaking or forming a bond, which is commonly used to indicate the stability of a molecule^36^. The theoretical value could be calculated using the B3LYP method in Density Functional Theory (DFT)^41^. It had an advantage of providing a simple and quick predictive on the potential for the new structural stability. However, at present, there are few studies on the characterisation of stability by DFT, and the scholars mainly focus on the calculation of OH bond energy in phenolic anti-oxidants^41–44^. The traditional methods for stability measure are generally performed by chromatography (HPLC, TLC, GC, and HPTLC), spectroscopy (UV, IR, and NMR) or a combination of that two (GC-MS, LC-MS, and LC-NMR)^45^. In this article, we creatively combined theoretical calculations with experiments to predict the stability of curcumin and dicarbonyl curcumin skeleton as well as the analogues with the same curcumin substituent group at the molecular level. Then, the stability of the compounds in PBS solution and target compound B2 in the cell culture system were determined by an ultraviolet spectrophotometer. Though the verification of the theoretical and experiments, it was more accurately explaining the actual stability of the compounds. Besides, it was also innovatively found that B2 could exert promising anti-tumour activity by inducing cell apoptosis and pyroptosis through ROS-mediated ER stress activation.

Pyroptosis is a form of programmed inflammatory cell death in which cells swell until their membranes rupture, resulting in the explosive release of cell contents that triggers a storm of intense inflammatory responses^46^. Recently, it has gradually realised that except acting as a significant defence mechanism in the inflammatory response, inducing cell pyroptosis is expected to be a new mechanism for tumour therapy^47^. In ovarian cancer, it is found that long non-coding RNA growth arrest-specific transcript 5 (lncRNA-Gas5) could act as an inducer of cell pyroptosis through activating caspase-1, IL-1β^48^. In gastric cancer, the NF-κB signalling pathway could be activated after helicobacter pylori infection, which upregulates the expression of IL-1, IL-6, TNF-α, and endothelial growth factor, thereby activating the inflammasome and triggering cell pyroptosis^49^. For lung cancer, the knock-down of LncRNA-XIST inhibits tumour progression by triggering miR-335/SOD2/ROS signal pathway mediated pyroptotic cell death^50^. Likewise, inducing cell pyroptosis is a new potential treatment for cervical cancer, breast cancer, malignant mesothelioma, etc^47^. In addition, the search for suitable conditions of pyroptosis will provide new references for tumour treatment and anti-cancer drug screening. Chemotherapeutic drugs have always played an important role in the treatment of tumours. In 2017, it was first reported in *Nature* that it also could induce cell pyroptosis^21^. For example, topotecan, irinotecan, etoposide, and cisplatin induced the death of Jurkat and MeWo cells through this mechanism^21^. Adriamycin and fluorouracil induced cell pyroptosis in HeLa cells^21^. Other studies have shown that cisplatin and paclitaxel through the pathway of caspase-3-GSDME to induce pyroptosis in lung cancer cells^51^. However, at present, it has only been reported that chemotherapeutic drugs induced cell pyroptosis in lung cancer by the NF-κB pathway, ROS inducing, APE1 target as well as NLRP3 inflammasome^52–55^, and much remains unknown about the regulatory mechanisms. Furthermore, there are few reports about the curcumin analogues and ROS-induced switch of apoptosis to pyroptosis. As reported, ROS is widely associated with ER stress^27^^,^^28^. Under normal circumstances, the stability of the ER environment is a guarantee for the normal function of ER. As a protective measure at the subcellular level, ER stress reduces the biosynthesis of protein and increased the degradation of ER by inducing URP in the early stages. With the intensification of ER stress, the activation of UPR would then be inhibited, causing the signal of survival switching to apoptosis. At present, the activation of ER stress is also gradually found involved in the cell pyroptosis in inflammation^56^. At the same time, Le et al. found that down-regulation of ZDHHC1 inhibited tumour growth by inducing oxidative/ER stress to induce cell apoptosis and pyroptosis^57^. However, this pathway has not been confirmed in lung cancer and there are no reports on chemotherapeutic drugs for inducing cell pyroptosis based on ROS/ER stress pathway. Thus, the relation of ROS-mediated ER stress and pyroptosis on lung cancer require to do in-depth study. In this article, we found that B2 could also induce cell pyroptosis and unlikely the most other reports on curcumin analogues and pyroptosis, it induced the switch from apoptosis to pyroptosis by up-regulating ROS. In addition, it was an innovative finding that ROS/ER signal axis mediated pyroptotic cell death in lung cancer and the compound B2 that both with great stability and anti-tumour activity through such new mechanism was expected to be a promising drug candidate.

## 5. Conclusions

In summary, in this article, two classes of dicarbonyl curcumin analogues were designed, and the rationality of the design was demonstrated by physicochemical calculations. The inhibitory activity of B series compounds on two kinds of tumour cells is better than that of A series compounds, and the inhibitory effect on H460 cells is more obvious. This provides a better template for the further design of anti-tumour compounds. Among these compounds, B2 with excellent anti-tumour activity for both two cell lines was screened out. Comparing with curcumin, the stability of B2 in phosphate buffer was significantly improved. B2 could also maintain a high concentration in H460 cells in comparison to curcumin, which was helpful to its anti-tumour activity. Moreover, it was demonstrated that B2 could induce cell apoptosis and pyroptosis, and thereby exerted anti-tumour effect. The emergence of apoptosis and pyroptosis was associated with ROS-mediated ER stress activation. These results indicated that B2 was a potential anti-tumour candidate drug.
